# Increased Systemic and Local Interleukin 9 Levels in Patients with Carotid and Coronary Atherosclerosis

**DOI:** 10.1371/journal.pone.0072769

**Published:** 2013-08-30

**Authors:** Ida Gregersen, Mona Skjelland, Sverre Holm, Kirsten B. Holven, Kirsten Krogh-Sørensen, David Russell, Erik T. Askevold, Christen P. Dahl, Stein Ørn, Lars Gullestad, Tom E. Mollnes, Thor Ueland, Pål Aukrust, Bente Halvorsen

**Affiliations:** 1 Research Institute of Internal Medicine, Oslo University Hospital Rikshospitalet, Oslo, Norway; 2 Faculty of Medicine, University of Oslo, Oslo, Norway; 3 Department of Neurology, Oslo University Hospital Rikshospitalet, Oslo, Norway; 4 Department of Nutrition, Institute for Basic Medical Sciences, Faculty of Medicine University of Oslo, Oslo, Norway; 5 Department of Thoracic and Cardiovascular Surgery, Oslo University Hospital Rikshospitalet, Oslo, Norway; 6 Department of Cardiology, Oslo University Hospital Rikshospitalet, Oslo, Norway; 7 K.G. Jebsen Cardiac Research Centre and Center for Heart Failure Research, Faculty of Medicine, University of Oslo, Oslo, Norway; 8 Division of Cardiology, Stavanger University Hospital, Stavanger, Norway; 9 Research Laboratory, Somatic Research Centre, Nordland Hospital, Bodø, University of Tromsø, Tromsø, Norway; 10 Department of Immunology, Oslo University Hospital Rikshospitalet, Oslo, Norway; 11 Section of Clinical Immunology and Infectious Diseases, Oslo University Hospital Rikshospitalet, Oslo, Norway; Massachusetts General Hospital and Harvard Medical School, United States of America

## Abstract

**Objective:**

Atherosclerosis is a chronic inflammatory disorder that involves a range of inflammatory mediators. Although interleukin (IL)-9 has been related to inflammation, there are at present no data on its role in atherosclerosis. Here we have examined IL-9 and IL-9 receptor (IL-9R) systemically and locally in patients with coronary and carotid atherosclerosis.

**Methods:**

Plasma IL-9 was quantified by enzyme immunoassay and multiplex technology. IL-9 and IL-9R mRNA were quantified by real-time RT-PCR, and their localization within the lesion was assessed by immunohistochemistry.

**Results:**

The main findings were: (i) Patients with carotid atherosclerosis had significantly raised IL-9 plasma levels compared with healthy controls (n = 28), with no differences between asymptomatic (n = 56) and symptomatic (n = 88) patients. (ii) On admission, patients with acute ST-elevation myocardial infarction (STEMI) (n = 42) had markedly raised IL-9 plasma levels which gradually declined during the first week post-MI. (iii) T cells and monocytes from patients with unstable angina (n = 17) had increased mRNA levels of IL-9 as compared with controls (n = 11). (iv) Carotid plaques (n = 68) showed increased mRNA levels of IL-9 and IL-9R compared to non-atherosclerotic vessels (n = 10). Co-localization to T cells (IL-9 and IL-9R) and macrophages (IL-9) were shown by immunohistochemistry. (v) IL-9 increased IL-17 release in peripheral blood mononuclear cells from patients with unstable angina (n = 5) and healthy controls (n = 5) with a particularly enhancing effect in cells from the patient group.

**Conclusion:**

Our findings show increased IL-9 levels in different atherosclerotic disorders both systemically and within the lesion, suggesting a role for the IL-9/IL-9R axis in the atherosclerotic process, potentially involving IL-17 mediated mechanisms. However, the functional consequences of these findings should be further investigated.

## Introduction

Complications from atherosclerosis are the leading causes of death in the Western world. Atherosclerotic plaque development is now regarded as a chronic inflammatory process which involves interactions between lipids, immune cells and the artery wall [1]. The inflammatory arm of atherosclerosis involves both adaptive and innate immune responses, and may be a common link for many atherosclerotic risk factors (e.g., obesity, hyperlipidemia and smoking) [2]. The small cell-regulatory proteins, called cytokines, are key players in this inflammatory network. Several studies demonstrate altered levels of various cytokines in atherosclerotic disorders, both systemically and within the atherosclerotic plaque [3]. However, although the concept of inflammation as a major mediator in atherosclerosis is well established, the identification of the different actors in this complex network is not fulfilled.

Interleukin (IL)-9 was first recognized as a T helper cell type 2 (Th2)-related cytokine, but it is now clear that IL-9 is produced by various CD4^+^ T-cell subsets, including the newly defined Th9 cells [4]. IL-9 expression is also observed in granulocytes, dendritic cells and mast cells [5]–[8]. The IL-9 receptor (IL-9R) consists of a specific α-chain and a signal unit (γ-chain) that is shared with other members of the IL-2-related cytokine family such as IL-2, IL-4, IL-7 and IL-15. While IL-9 has been implicated as a pathogenic mediator of asthma and allergic disorders, recent studies suggest that IL-9 may enhance as well as dampen the progression of various autoimmune disorders [9]–[12]. Increased plasma levels of IL-9 are seen in patients with heart failure, possibly correlated to disease progression [13], and increased IL-9 levels have been reported in experimental cardiomyopathy [14]. IL-9 has also been shown to facilitate Th17 cell expansion, and to stimulate production of IL-17 from mononuclear cells of both healthy and diseased subjects such as patients with psoriasis [15]. To this end, however, there are to the best of our knowledge, no data on the regulation of IL-9 in atherosclerotic disorders.

Based on its known association with other inflammatory conditions, we hypothesized that IL-9 could be involved in the inflammatory arm of atherosclerotic disease. We examined this hypothesis by various experimental approaches including studies on the expression of IL-9 and IL-9R in patients with carotid and coronary atherosclerosis. We also assessed the ability of IL-9 to modulate inflammatory responses with relation to atherosclerotic disorders *in vitro*.

## Materials and Methods

### Ethics

The study protocols were approved by the Regional Health Authorities of South-Eastern and Western Norway. The study confirms with the principles outlined in the Declaration of Helsinki for use of human tissue or subjects. Signed informed consent was obtained from all participants.

### Patients and Control Subjects

Three populations of patients with atherosclerotic disorders were investigated. (**i**) *Patients with carotid atherosclerosis.* Patients (n = 144) with high-grade internal carotid stenosis (≥70%) treated with endarterectomy or carotid angioplasty with stenting were included in the study (Table S1). The patients were classified as asymptomatic (n = 56) or symptomatic (n = 88) in accordance to absence or presence of clinical symptoms (i.e., stroke, transient ischemic attack (TIA) or amaurosis fugax ipsilateral to the stenotic internal carotid artery within the past 6 months). Carotid stenoses were diagnosed and classified by precerebral color Duplex ultrasound and CT angiography according to consensus criteria [16], [17]. The asymptomatic carotid stenoses were detected during clinical examinations of patients with coronary artery disease (CAD), peripheral artery disease or stroke/TIA more than 6 months previously. For comparison, plasma was collected from 28 sex- and age-matched healthy subjects (Table S1). (**ii**) *Patients with ST segment elevation myocardial infarction (STEMI).* Plasma samples from 42 patients with first time STEMI were obtained at admission, immediately prior to percutaneous coronary intervention (PCI) and 2, 7 and 60 days following hospitalization (Table S2). Median time from symptom debut to admission was 145 minutes (range: 25–720 minutes). The study design has previously been described [18] and patients were included if they had (i) no previous MI, (ii) demonstrated acute proximal/mid-occluded single vessel disease and (iii) underwent successful PCI with stent implantation without significant residual stenosis. For comparison, plasma was collected from 10 sex- and age-matched healthy subjects. (**iii**) *Patients with stable and unstable angina.* IL-9 and IL-9R mRNA expression were examined in T cells and monocytes from 30 CAD patients and 11 sex- and age-matched healthy subjects. The patient population consisted of 13 patients with stable and 17 patients with unstable angina. Patients with unstable angina were characterized by chest pains at rest within the preceding 48 hours with transient ST-T segment depression and/or T-wave inversion (i.e., Braunwald’s class IIIBa), but with no evidence of myocardial necrosis by enzymatic criteria. Patients with stable CAD showed stable effort angina with over 6 month duration and a positive exercise test. All patients had angiographically documented obstruction (≥50%) of at least one main coronary artery. Exclusion criteria were MI or thrombolytic therapy in the previous month.

In all sub-studies, patients with contaminant inflammatory diseases, malignancies or overt liver and kidney disease were excluded.

### Blood Sampling Protocol

Blood samples from all patients and controls were drawn into pyrogen-free EDTA tubes, immediately placed on ice, and centrifuged at 2500*g* for 25 minutes within 20 minutes to obtain platelet-poor plasma. Plasma was stored at −80°C until analyses. Samples were thawed less than three times.

### Tissue Sampling from Carotid Plaque

Atherosclerotic carotid plaques were collected during carotid endarterectomy. Plaques that were used for RNA extraction were rapidly frozen in liquid nitrogen. Plaques that were used for immunohistochemistry (IHC) were placed in 4% phosphate buffered-formalin for 48 hours and then embedded in paraffin. For mRNA analyses samples from the common iliac artery of deceased organ donors were used as non-atherosclerotic vessel controls. Control tissues were prepared and stored in the same way as carotid plaques.

### Isolation of Leukocyte Subsets

Peripheral blood mononuclear cells (PBMCs) were separated from heparinized blood by Isopaque-Ficoll gradient centrifugation using Lymphoprep (Nycomed, Oslo, Norway). PBMCs were immediately used for *in vitro* experiments or for further separation of CD14^+^ monocytes (CD14-labeled magnetic beads; MACS, Milteny Biotec, Bergisch Gladbach, Germany) and CD3^+^ T-cells (negative selection by monodisperse immunomagnetic beads; Dynal, Oslo, Norway) as described elsewhere [19], [20]. After isolation, the cells were immediately stored in liquid nitrogen. The selected T cells consisted of >90% CD3^+^ cells and the isolated monocytes of >95% CD14^+^ cells as assessed by flow cytometry.

### Immunohistochemistry

Sections (5 µm) of paraffin-embedded atherosclerotic vessels were treated with 0.5% H_2_0_2_, followed by high-temperature unmasking in citrate-buffer (pH 6.0), blocked with 0.5% bovine serum albumin (BSA) and then incubated with primary antibodies (anti-IL-9; Santa Cruz Biotechnology, CA and anti-IL-9R; Abcam, Cambridge, UK) for one hour at room temperature. After washing, the slides were incubated for an additional 30 minutes with peroxidase-conjugated secondary antibodies (Impress-Vector, Vector laboratories, Burlingame, CA), rinsed and developed with chromogen for immunoperoxidase staining (DAB Plus, Vector laboratories) for seven minutes. The sections were counterstained with Hematoxylin. Omission of the primary antibody served as a negative control.

### Immunofluorescence

Paraffin-embedded sections (5 µm) of atherosclerotic carotid plaques were exposed to high-temperature unmasking (citrate-buffer, pH 6.0), blocked in 0.5% BSA and incubated over night at 4°C with primary antibodies (anti-IL-9; Santa Cruz Biotechnology, CA, anti-IL-9R; Abcam, Cambridge, UK, anti-CD68; DAKO, Glostrup, Denmark and anti-CD3; Santa Cruz Biotechnology). The sections were counterstained with secondary antibodies, Alexa Fluor 488 and 568 (both from Invitrogen, Eugene, OR). Nuclei were stained with diamidino-2-phenylindole (DAPI) (Slow Fade Gold antifade reagent, Invitrogen). Fluorescent images were obtained on a Nikon Eclipse E400 microscope with 400× magnification.

### Cell Culture Experiments

Freshly isolated PBMCs were incubated in flat-bottomed 24-well microtitre plates (2×10^6^/ml, 500 µl/well; Costar, Sigma-Aldrich, St. Louis, MO) with RPMI 1640 medium (PAA Laboratories, Pasching, Austria) supplemented with 10% heat inactivated human AB^+^ serum (Invitrogen, Carlsbad, CA) and 1% Penicillin-Streptomycin (Sigma). The cells were stimulated with recombinant human IL-9 (100 ng/ml, R&D Systems, Minneapolis, MN) with and without phytohaemagglutinin (PHA, 20 µg/ml; Thermo Scientific, Waltham, MA). Cell-free supernatants were harvested after 24, 48 and 72 hours and stored at −80°C.

### Measurements of Cytokines

In patients with carotid atherosclerosis and corresponding controls, plasma levels of IL-9 was quantified by enzyme immunoassay (EIA) obtained from eBioscience (San Diego, CA). In STEMI patients and corresponding controls, plasma levels of IL-9 was quantified by multiplex cytokine immunoassay (Bio-Plex, Bio-Rad Laboratories, Hercules, CA), analyzed on a Multiplex Analyzer (Bio-Rad Laboratories). In the *in vitro*-experiments, IL-17AF, hereafter termed IL-17, was analyzed in supernatants by EIA (R&D Systems).

### Real-time Quantitative RT-PCR

Total RNA was obtained from atherosclerotic and non-atherosclerotic vessels and from T cell and monocytes with the use of RNeasy spin columns (QIAGEN, Hilden, Germany). All samples were subjected to DNase treatment (RQI DNase; Promega, Madison, WI) and stored at −80°C until further analysis. cDNA synthesis was performed using the High-Capacity cDNA Reverse Transcriptation Kit (Applied Biosystems, Foster City, CA). Gene expression was examined by real-time quantitative RT-PCR (7500 Fast Real-Time PCR System, Applied Biosystems). Sequence specific TaqMan primers and probes were used for IL-9R detection (Assay-ID: HS_00602538_M1, Applied Biosystems). mRNA detection of IL-9 and the reference genes β-actin and 18S RNA were assessed with SyberGreen primers designed using Primer Express software 3.0 (Applied Biosystems): (IL-9: forward primer [FP] 5′-AGTGCAGTGGTGCCATCTGA-3′ and reverse primer [RP]: 5′-ACGCACCTGTAATGCCAGCTA-3′), β-actin: (FP 5′-AGGCACCAGGGCGTGAT-3′ and RP 5′-TCGTCCCAGTTGGTGACGAT-3′) and 18S (FP 5′-CGGCTACCACATCCAAGGAA-3′ and RP 5′-GCTGGAATTACCGCGGCT-3′). The relative mRNA level of each transcript was calculated by the ΔΔCt-method.

### Statistical Analysis

For unpaired data the Mann-Whitney U-test and Kruskal-Wallis test were used when appropriate. For paired data the paired t-test or Wilcoxon signed rank test was used. The chi-square and Fischer’s exact test were used for categorical data. Statistical significance was considered at p<0.05 for all data.

## Results

### Increased Circulating Levels of IL-9 in Carotid Atherosclerosis

**Figure 1:**
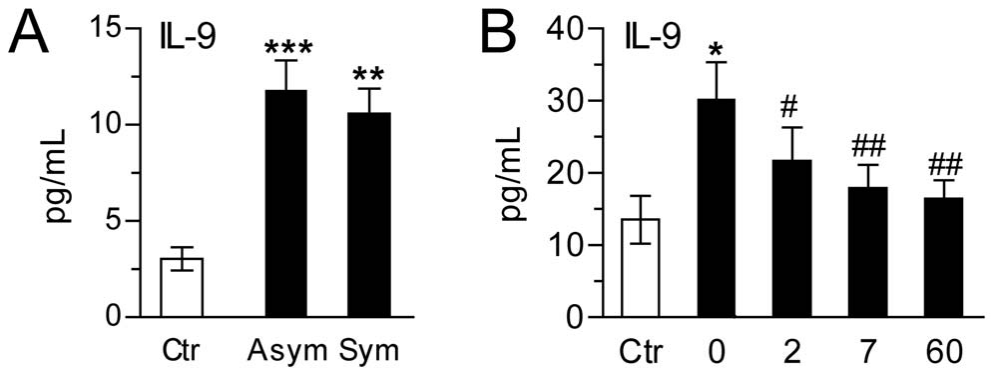
Increased plasma levels of IL-9 in patients with carotid and coronary atherosclerosis. Panel **A** shows plasma levels of IL-9 in patients with asymptomatic (n = 56) and symptomatic (n = 88) carotid plaques and in healthy controls (n = 28). Panel **B** shows plasma levels of IL-9 in patients with STEMI (n = 42) at admission and at different time points after PCI (2, 7 and 60 days). For comparison, levels were also measured in healthy controls (n = 10). Data are presented as mean±SEM. *p<0.05, **p<0.01 and ***p<0.0001 versus controls. #p<0.05 and ##p<0.01 versus levels at admission.

Baseline characteristics of the patients with carotid atherosclerosis are shown in Table S1. Both asymptomatic and symptomatic patients had significantly higher plasma levels of IL-9 than healthy controls, with no significant difference between the two patient groups (Fig. 1A). In the patient group as a whole, we found no significant correlation between IL-9 levels and any of the variables outlined in Table S1.

### Increased Circulating Levels of IL-9 in Coronary Atherosclerosis

We next examined circulating IL-9 levels in a STEMI population, representing another atherosclerotic disorder (Table S2). Plasma IL-9 was significantly elevated in these patients at admission compared to healthy controls (Fig. 1B). From 48 hours following PCI, there was a gradual decline in IL-9, almost reaching normal levels during the end of the observation period (Day 60). Prior to PCI no correlation between IL-9 and CRP or maximum troponin T was observed for these patients.

### Increased Expression of IL-9 and IL-9R in CD3^+^ T cells and CD14^+^ Monocytes in Patients with Coronary Atherosclerosis

**Figure 2:**
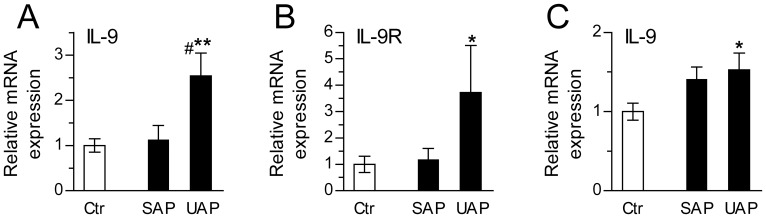
Increased expression of IL-9 and IL-9R in immune cells from patients with coronary atherosclerosis. mRNA levels of IL-9 and IL-9R were quantified by real-time RT-PCR in CD3^+^ T cells (**A** and **B**) and in monocytes (**C**) from healthy controls (n = 11) and from patients with stable (SAP, n = 11) and unstable (UAP, n = 17) angina. mRNA levels are related to the reference gene 18S (T cells) and β-actin (monocytes) and normalized to levels in healthy controls. For monocyte analyses, only samples from 9 of the controls and 13 of the patients with unstable angina were available. No IL-9R transcripts were detected in patients or controls in these cells. Bars represent mean±SEM. #p<0.05 versus stable angina, *p<0.05 and **p<0.01 versus controls.

To further examine the regulation of IL-9 in atherosclerotic disorders, we analyzed mRNA levels of IL-9 and IL-9R in T cells and monocytes from patients with stable and unstable angina and in healthy controls. T cells from patients with unstable, but not stable angina had significantly increased mRNA expression of IL-9 and IL-9R compared with T cells from healthy controls, and for IL-9, the difference between unstable and stable disease was also significant (Fig. 2A–B). The same pattern was seen for IL-9 in monocytes with increased mRNA levels in unstable, but not in patients with stable angina compared to healthy controls (Fig. 2C). IL-9R transcripts were not detected in monocytes from patients or controls.

### Increased Expression of IL-9 and IL-9R in Atherosclerotic Carotid Plaques

**Figure 3:**
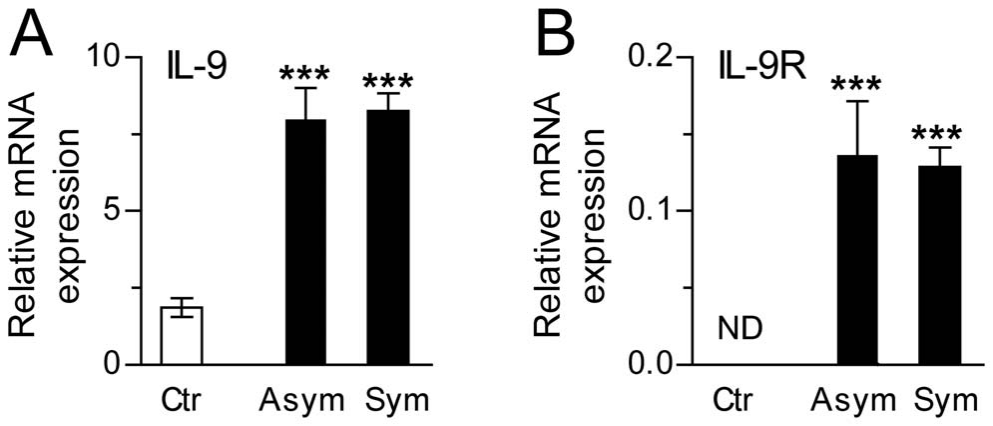
Increased expression of IL-9 and IL-9R in human atherosclerotic carotid plaques. mRNA levels of IL-9 (**A**) and IL-9R (**B**) in patients with asymptomatic (n = 13) and symptomatic (n = 55) carotid stenosis and in non-atherosclerotic vessels obtained from organ donors (common iliac artery, n = 10) were quantified by real-time RT-PCR. No IL-9R transcripts were detected in control samples. Levels of IL-9 and IL-9R expression are related to reference gene β-actin. Data are presented as mean±SEM. ***p<0.0001 versus controls.

**Figure 4:**
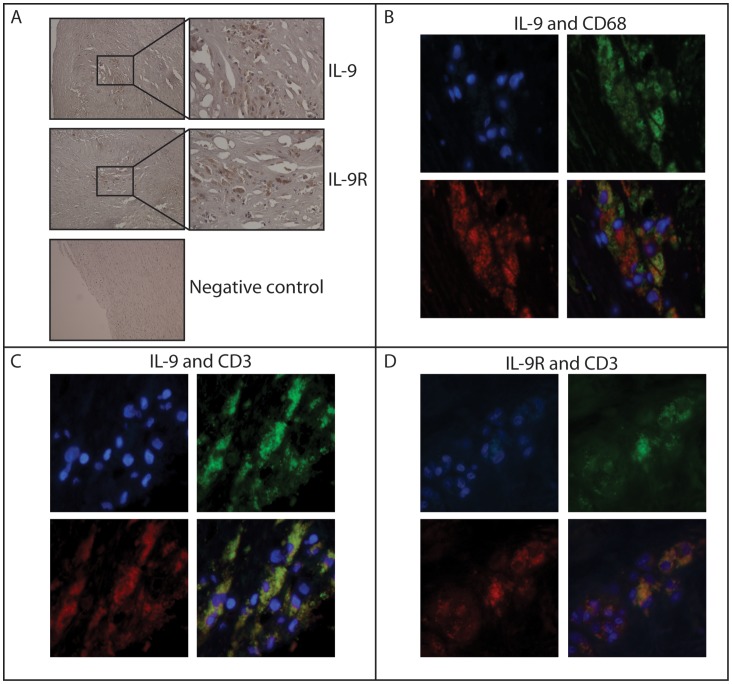
Localization of IL-9 in human carotid atherosclerotic plaques. Panel **A** shows immunostaining of IL-9 and IL-9R in symptomatic carotid atherosclerotic plaques. Representative images obtained with 100× and 400× objective. Omission of primary antibody is shown as a negative control. Panel **B** shows double immunofluorescent staining of IL-9 (red fluorescence), CD68 (macrophages, green fluorescence) and nucleus (DAPI, blue fluorescence) from symptomatic carotid atherosclerotic plaques. The bottom right picture is a merge of the three. Panel **C** shows staining of IL-9 (red) and CD3 (T-cells, green), nucleus (blue) and merge. Panel **D** shows staining of IL-9R (red) and CD3 (T-cells, green), nucleus (blue) and merge. The pictures are representatives of multiple staining (n = 5–8).

The findings so far suggested increased systemic expression of IL-9 in both carotid and coronary atherosclerosis. We therefore examined whether increased IL-9/IL-9R levels could be found within the atherosclerotic lesion. Carotid lesion samples from patients with asymptomatic and symptomatic carotid atherosclerosis had markedly increased expression of IL-9 and IL-9R as compared with samples from non-atherosclerotic vessels (common iliac artery, Fig. 3). In fact, while IL-9 mRNA was expressed in both controls and patients, transcript of IL-9R could not be detected in control samples (Fig. 3B). There were, however, no differences between the two patient groups for either IL-9 or IL-9R. Moreover, although markedly raised as compared with non-atherosclerotic vessels, the transcript levels of IL-9 and IL-9R within the lesion were in general low. Immunostaining confirmed the expression of IL-9 and IL-9R within the plaque, and co-localization with T cells (IL-9, IL-9R) and macrophages (IL-9) was supported by co-staining to the respective cell markers, CD3 and CD68 (Fig. 4).

### IL-9 Promotes IL-17 Release from PBMCs

**Figure 5:**
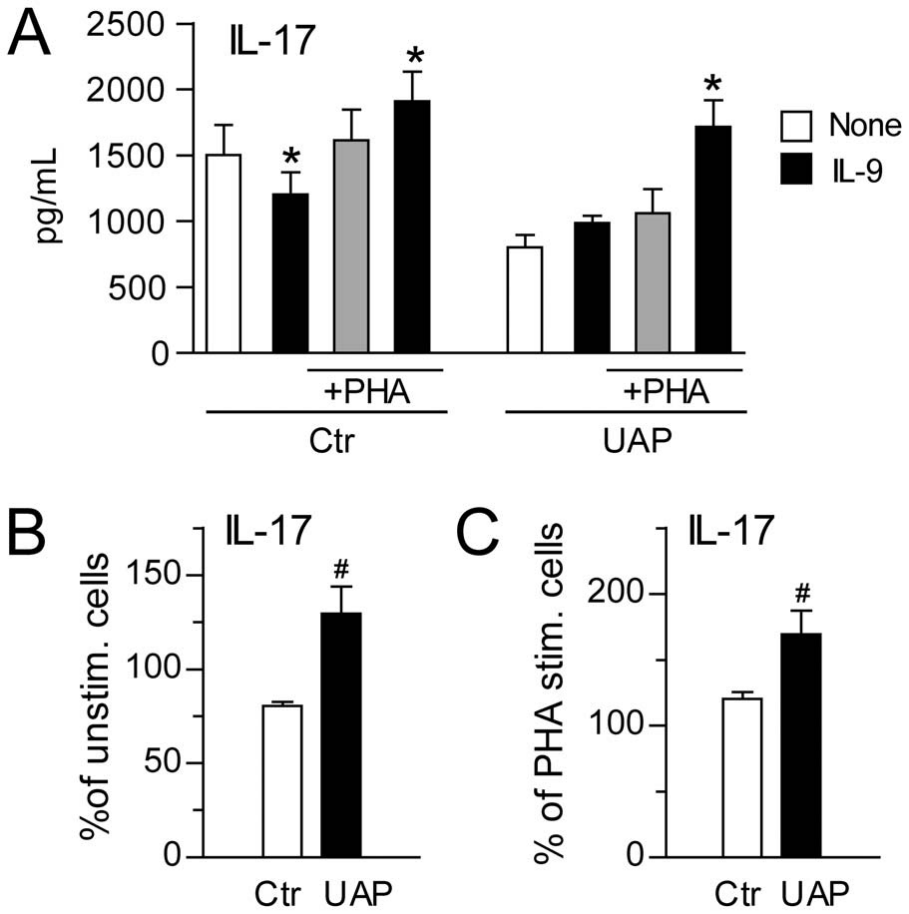
IL-9 promotes IL-17 release in PBMCs. PBMCs from healthy controls (Ctr, n = 5) and patients with unstable angina (UAP, n = 5) were stimulated with IL-9 (100 ng/ml) with and without co-stimulation with PHA (20 µg/ml). Panel **A** shows the absolute release of IL-17 after culturing for 72 hours as assessed by EIA measurements in cell-free supernatants (controls to the left, patients to the right). Panel **B** shows the percentage change in IL-17 release when adding IL-9 to unstimulated cells from healthy controls (left) and patients (right). Panel **C** shows the percentage change in IL-17 release when adding IL-9 to PHA-stimulated cells from healthy controls (left) and patients (right). Data are given as mean±SEM. *p<0.05 versus comparative condition without IL-9 (unstimulated and PHA stimulated, respectively). #p<0.05 versus healthy controls.

IL-9 has previously been reported to alter production of both pro- and anti-inflammatory mediators in leukocyte subsets with promotion of Th17 cells as one of its features [15], [21]–[23]. To examine any potential consequences of the increased IL-9 levels in atherosclerotic disorders, we therefore next examined the effects of IL-9 (100 ng/ml) on IL-17 release in PBMCs from patients with unstable angina (n = 5) and healthy controls (n = 5), with and without co-stimulation with PHA (20 µg/ml). IL-9 induced a significant increase in IL-17 release in PHA activated PBMCs after culturing for 72 hours in both patients and controls, with a particularly enhancing effect in the patient group (Fig. 5). While IL-9 when given alone induced a significant decrease of IL-17 in PBMCs from healthy controls, there was a modest increase in IL-17 from patient cells, resulting in a significant difference in percentage change in the IL-9-induced IL-17 release between patients and controls (Fig. 5A–B).

## Discussion

Here we report for the first time increased levels of both IL-9 and IL-9R in patients with carotid and coronary atherosclerosis. Through investigation of three different populations of atherosclerotic patients we found elevated circulating levels of IL-9 and increased expression in cells and tissue of IL-9 and IL-9R, compared to healthy controls.

IL-9 has previously been associated with inflammatory diseases in several studies, especially in relation to allergies and asthma. There are also some reports of increased IL-9 levels in relation to systemic sclerosis and experimental transplant allograft rejection [10]–[12], [24]. However, the role of IL-9 in cardiovascular disorders is largely unknown. Increased plasma IL-9 has been reported in patients with ischemic and non-ischemic cardiomyopathies, inversely correlated with parameters of impaired left ventricular function [13], and increased serum IL-9 has also been reported in 45 patients with acute ischemic stroke of different etiology [25]. However, the present study is, to the best of our knowledge, the first report of increased levels of IL-9 in patients with confirmed coronary and carotid atherosclerosis. We found increased plasma levels of IL-9 in patients with carotid atherosclerosis with no differences between asymptomatic and symptomatic patients. Moreover, patients with STEMI had markedly elevated IL-9 levels on admission, a few hours after symptom debut, with a gradual decline during the following week. In contrast to several other inflammatory markers [18], [26], [27], there was no IL-9 increase following PCI, suggesting only minor regulation by ischemia and reperfusion injury. Finally, enhanced expression was also seen at the cellular level with increased levels of IL-9 mRNA and IL-9R in T cells from patients with unstable angina, and for IL-9, this up-regulation was also seen in monocytes.

In addition to increased systemic expression, the present study also showed enhanced expression of IL-9 and its receptor within the atherosclerotic lesion. Thus, when compared with non-atherosclerotic vessels (common iliac artery), carotid plaques had markedly elevated mRNA levels of IL-9 and IL-9R. Moreover IL-9 co-localized to both T cells and macrophages and IL-9R to T-cells, as assessed by IHC. The fact that IL-9 and IL-9R showed the same pattern in asymptomatic and symptomatic lesions could suggest that these mediators are related to the chronic atherosclerotic process, rather than to plaque destabilization.

IL-9 has been shown to induce cytokine release from activated mast cells, and IL-9 is also suggested to function as an autocrine growth factor that facilitates the expansion of inflammatory Th17 cells [10], [22], [28]. Indeed, in the present study we found that IL-9 promoted a IL-17 release in PBMCs, with a particularly marked response in cells from unstable angina patients. In fact, while IL-9 seemed to suppress IL-17 release in PBMCs from healthy controls, it enhanced IL-17 release in cells from unstable angina patients. These new findings further underscore a link between IL-9 and IL-17 and suggest that this link is particularly strong in patients with unstable angina, potentially reflecting up-regulation of IL-9R in T cells from these patients. However, IL-9 may also possess anti-inflammatory properties by enhancing the function of regulatory T cells [22] and by suppressing IL-12 production in certain antigen presenting cells [29]. Moreover, of relevance to stroke, IL-9 has been shown to protect cortical neuron from cell death by anti-apoptotic mechanisms [30], and further studies are clearly needed to elucidate the net effect of IL-9 levels in atherosclerotic disorders.

In this study we demonstrate increased levels of IL-9, as well as increased expression of IL-9R, both systemically and within the lesion of patients with atherosclerotic disorders. This suggests a role for the IL-9/IL-9R interaction in atherosclerosis, but the functional consequences of these findings are at present unclear.

## Supporting Information

Table S1 Characteristics of patients with carotid artery disease and controls.
(DOC)

Table S2 Characteristics of patients with STEMI.
(DOC)
