# Supplementary material for: Increased Systemic and Local Interleukin 9 Levels in Patients with Carotid and Coronary Atherosclerosis
Source: PLoS One. 2013 Aug 30;8(8):e72769. doi: 10.1371/journal.pone.0072769 (PMC3758349; doi:10.1371/journal.pone.0072769)
Supplement: Table S1 — Characteristics of patients with carotid artery disease and controls. (DOC) [file pone.0072769.s001.doc]

**Table S1.**

|  | | Controls  (n=28) | Asymptomatic patients  (n=56) | Symptomatic patients  (n=88) | | p-value |
| --- | --- | --- | --- | --- | --- | --- |
| Age, years | | 58±2 | 66±1 | | 67±1 | 0.371 |
| Gender, F/M | | 9/19 | 17/39 | | 33/55 | 0.380 |
| BMI, kg/m^2^ | | 24.5±0.5 | 26.5±0.6 | | 25.7±0.5 | 0.265 |
| Smokers, % | | 17 | 49 | | 57 | 0.618 |
| Hypertension,%* | | - | 64 | | 64 | 0.879 |
| Diabetes, % | | - | 13 | | 16 | 0.623 |
| CRP, mg/l | | 1.2±0.2 | 6.9±1.4 | | 5.6±0.8 | 0.388 |
| Medication, % | |  |  | |  |  |
|  | Aspirin | - | 79 | | 85 | 0.424 |
|  | Statin | - | 84 | | 86 | 0.956 |
| Total cholesterol, mmol/l | | 6.1±0.2 | 4.2±0.1 | | 4.4±1.2 | 0.346 |
| LDL cholesterol, mmol/l | | 3.9±0.2 | 2.5±0.1 | | 2.7±0.2 | 0.319 |
| HDL cholesterol, mmol/l | | 1.7±0.1 | 1.3±0.1 | | 1.3±0.1 | 0.708 |
| Triglycerides, mmol/l | | 1.0±0.1 | 1.5±0.1 | | 1.5±0.1 | 0.931 |
| Grade of stenosis %  Echolucent plaque, % | | -  - | 80.4±1.7  20 | | 81.2±1.1  34 | 0.318  0.091 |

Data are mean±SEM or percentage of group (%). Gender is presented as values. F, female; M, male; BMI, body mass index; CRP, C reactive protein; Statin, hydroxymethylglutaryl coenzyme A reductase inhibitor; LDL, low-density lipoprotein; HDL, high-density lipoprotein. *Hypertension is defined as use of antihypertensive medication. P-values indicate differences between asymptomatic and symptomatic patients
