# Supplementary material for: Increased Systemic and Local Interleukin 9 Levels in Patients with Carotid and Coronary Atherosclerosis
Source: PLoS One. 2013 Aug 30;8(8):e72769. doi: 10.1371/journal.pone.0072769 (PMC3758349; doi:10.1371/journal.pone.0072769)
Supplement: Table S2 — Characteristics of patients with STEMI. (DOC) [file pone.0072769.s002.doc]

**Table S2.**

|  | | | n=42 |
| --- | --- | --- | --- |
| Age (years) | | | 58 ±2 |
| Gender, F/M | | | 8/34 |
| Smokers, % | | | 48 |
| Hypertension, % | | | 24 |
| Diabetes, % | | | 7 |
| Medications, % | |  | |
|  | Gp IIb/IIIa antagonist. | | 74 |
|  | Heparin | | 100 |
|  | Clopidogrel | | 100 |
|  | Aspirin | | 100 |
|  | Statin | | 100 |
|  | ACEI/ARB | | 91 |
|  | Beta blocker | | 55 |
|  | Aldosterone antagonist. | | 19 |

Data are shown as mean±SEM or percentage of group (%). F, female; M, male; Statin, hydroxymethylglutaryl coenzyme A reductase inhibitor; ACEI, angiotensin-converting enzyme inhibitor; ARB, angiotensin receptor blocker.
